# Sustained Elimination of Iodine Deficiency Within the Third Decade After Compulsory Iodine Supplementation Policy in the South of Iran: A Population-Based Cross-Sectional Study

**DOI:** 10.1016/j.cdnut.2022.100013

**Published:** 2022-12-22

**Authors:** Marjan Jeddi, Ashkan Habib, Alireza Salehi

**Affiliations:** 1 Endocrinology and Metabolism Research Center, Shiraz University of Medical Sciences, Shiraz, Iran; 2 School of Medicine, Shiraz University of Medical Sciences, Shiraz, Iran; 3 MPH Department, School of Medicine, Shiraz University of Medical Sciences, Shiraz, Iran

**Keywords:** epidemiology, iodine deficiency, urine iodine, thyroid, Iran

## Abstract

**Objectives:**

Recently, some studies in Iran have shown mild to moderate iodine concentrations in adult and pregnant women populations despite iodine sufficiency in children. This study aimed to evaluate the urine iodine status and salt intake among adult households in the city of Sadra, Fars province in southern Iran, and to assess its possible influencing factors.

**Method:**

Participant households for this cross-sectional study were selected using randomized cluster sampling in the city of Sadra, Fars province, southern Iran from 1 February, 2021 to 30 November, 2021. Two subjects >18 y of age from each household were invited. Ninety-two subjects (24 men, 68 women) were enrolled. The participants were asked to collect their 24 h urine. They were then examined for thyroid disorders and subjected to thyroid ultrasonography and thyroid function tests. Urine samples were tested for iodine, sodium, and creatinine concentrations. Household salt intake was also estimated.

**Results:**

Median urine iodine content (UIC) in the participants was 175 (IQR: 117, 250) μg/L, whereas the median salt consumption per person per day was 9.6 (IQR, 7.3-14.5) g. Sex, methods of salt storage, presence of goiter or thyroid nodules, the addition of salt in the cooking stage, and subclinical hypothyroidism had no effect on UIC, whereas individuals with hypertension and lower education had significantly lower iodine concentrations. UIC had a significant positive correlation with urine sodium and thyroid stimulating hormones (TSH) concentrations (*P* < 0.001, 0.046) and a negative correlation with thyroid volume and T4 (*P* = 0.029, 0.018).

**Conclusion:**

Iodine status in the adult population of Sadra city was categorized as sufficient, although the iodine concentrations reported in Tehran were insufficient. The contributing factor can be higher salt consumption or possible higher environmental iodine concentrations in Sadra city than Tehran**.**

## Introduction

Iodine deficiency is the most prevalent preventable cause of mental disability [1]. It has long been recognized as a micronutrient essential for neural development and growth [2]. Iodine deficiency disorders (IDDs) can include symptoms such as hypothyroidism, goiter, spontaneous abortion, and cretinism. Chronic iodine deficiency has also been linked to follicular thyroid carcinoma [3]. Therefore, the maintenance of sufficient iodine concentrations in adults is crucial [1].

Epidemiological criteria for assessing iodine nutrition are based on urinary iodine concentrations of school children and pregnant women. Most iodine absorbed in the body eventually appears in the urine. Therefore, urinary iodine excretion is a good marker of recent dietary iodine intake [4]. Currently, World Health Organization (WHO) has recommended that urine specimens should be collected for urinary iodine assessment in school children and pregnant and lactating women during household-based surveys [5]. In children and non-pregnant women, median urinary iodine concentrations from 100 μg/L to 299 μg/L indicate that the individual has no iodine deficiency [5].

Despite global efforts to curb iodine deficiency through measures such as the universal salt iodization (USI) program, some previously iodine-sufficient countries have experienced a resurgence of iodine deficiency. Based on a report by the Iodine Global Network [6], countries such as Norway, Finland, and Germany have experienced iodine insufficiency in 2019 despite being previously classified as iodine sufficient. The United States has also seen a dramatic decrease in urine iodine concentrations from 1974 to 2010 [1].

In Iran, a salt iodization program was started in 1996 and iodized salt was reported to be the main dietary iodine source [7]. In 2013 data from the fifth national survey in Iran [8] showed that in all provinces, iodized salt consumption of households was 98%. In 2018 Shamsollahi and coworkers [7] showed that >80% of available salts in Iran have a suitable or acceptable concentration of iodine.

Some studies in Iran [9, 10] have shown mild to moderate iodine concentrations in adult and pregnant women populations despite iodine sufficiency in children [11]. Nazeri et al. [10] believe that the reduction of daily salt intake can contribute to the reduction of urine iodine concentrations, as demonstrated in their study. Accordingly, we designed this study to evaluate the urine iodine status and salt intake among adult households in Sadra city, Fars province, southern Iran, as well as to assess its possible influencing factors compared with the results of the study by Nazeri et al. [10] in Tehran.

## Methods

### Subjects

This cross-sectional study was performed in the city of Sadra, located 15 km northwest of Shiraz, Fars province, Iran. With a population of 122,226, it is the fourth most populous city in the province. Subjects were selected using cluster sampling. The city was divided into 2 clusters. In each cluster, 25 houses were randomly selected based on the postal code. In each house, the mother and a member aged >18 y, who did not meet the exclusion criteria, were selected. Data were collected from February 1, 2021 to November 30, 2021. Inclusion criteria were: age >18 y and residence in Sadra city. Exclusion criteria were: thyroid disorders or use of thyroid-related drugs, diuretic medications, pregnancy, or lactation. A data collection form [Supplementary Material] containing questions about gender, age, education level, thyroid disorder and related drug use history, blood pressure and related drug use history, salt container storage (in sunlight vs. dark and out of sunlight), and the cooking stage at which the salt was usually added (before cooking, during cooking, or after cooking) was used.

### Ethical review

Signed informed consent was taken from all participants, and the study was approved by the ethics committee of Shiraz University of Medical Sciences (Approval code: IR.SUMS.MED.REC.1398.568). The research assistant, a trained laboratory technician, explained the project to the participants and emphasized its benefits for the entire community. The subjects were asked to cooperate during various stages from completing the questionnaire to submitting blood and urine samples and performing an ultrasound. At each stage, if an abnormal test or ultrasound finding was detected, the individual was referred to an endocrinologist.

Caregivers were also asked for permission to use their data in future research. The results of the tests remained completely confidential and data analysis was done anonymously. The participants were not paid to participate but were reimbursed for travel to the study sites.

### Thyroid examination and salt sample collection

A preweighed container of iodized salt was given to each household to be exclusively used for 14 d, including during food preparation and in table salt shakers. Then, the amount of salt used was measured and divided by the number of people in each household. Participants were also examined and subjected to ultrasonography for goiter or thyroid nodules by an endocrinologist at the Endocrinology and Metabolism Research Center of the Shiraz University of Medical Sciences. Thyroid volume was calculated from the length, width, and depth of the thyroid gland using the formula: width × length × depth × π /6 [12].

### Urine and serum biochemical analysis

Urine samples over 24 h were taken from 2 residents of each household. Urine collection started with the second urine passed in the morning and ended with the first urine passed the following morning in a labeled 2.5 L plastic container. Subjects were also invited to undergo a blood test in the fasting state for assessing thyroid function including thyroid stimulating hormones (TSH), thyroxine (T4), and triiodothyronine (T3). Urine and blood analyses were performed before using the labeled salt package. Urine containers were sent to the Valfajr health center in Shiraz and stored at −20°C until analysis.

The UIC (μg/L) was measured using the Sandell-Kolthoff method as recommended by the WHO [5, 13]. Inter- and intra-assay coefficients of variation were 9.6% and 10.4%, respectively. The urine sodium level (mEq/L) was analyzed by emission flame photometry (Corning 480), in 50 participants with a coefficient of variation of 2%. Salt intake was estimated using urinary sodium excretion (1 g salt was equivalent to 17.1 mmol sodium) within 24 h in 50 study subjects [14].

Serum TSH was analyzed using an immunoradiometric assay (IRMA), whereas serum T4 and T3 were determined by radioimmunoassay (RIA) kits. Inter- and intra-assay coefficients of variation for TSH, T4, and T3 were 3.5%–2.4%, 4.5%–3.3%, and 3.8%–4.7%, respectively.

WHO guidelines [5] define a median adult UIC of >100 μg/L as sufficient. Therefore, UIC cutoffs intended for school children and extended to the general population by the WHO were used as a proxy. Median UIC values of <20 μg/L, 20–49 μg/L, 50–99 μg/L, and 100–199 μg/L were labeled as severe, moderate, and mild deficiency, and sufficiency, respectively [9]. The completion of 24-hour urine collection was assessed using urine creatinine concentrations. Urine creatinine values of <500 mg/d indicated incomplete urine collection and the corresponding samples were excluded.

### Statistical analysis

Data were analyzed using the statistical programs SPSS for statistics v. 25.0 (IBM) and STATA v. 17 (Stata Corp). UIC was not normally distributed. The median and interquartile range (IQR) of UIC concentrations were reported as recommended by WHO [5]. Quantitative variables between groups were compared using Mann-Whitney U and Kruskal-Wallis tests. Correlation between the variables was assessed using two-sided Spearman’s correlation coefficient. Predictors of UIC were identified using quantile regression (median regression). Statistical significance was determined at <0.05 with a confidence interval of 95%.

## Results

Ninety-two subjects (24 men, 68 women) were enrolled in this study. Their mean age was 40.2 ± 14.2 y. The average number of household members was 3.7 ± 1.1, whereas the average duration of stay in this city was 25.1 ± 16.5 y. The participants' level of education ranged from below high school (*N* = 50, 48.5%), high school graduate (*N* = 43, 41.7%), college graduate (*N* = 6, 5.7%), and advanced degree graduate (*N* = 1, 1%).

Median UIC in the population was 175 μg/L (IQR: 117–250 μg/L), the median urine sodium content was 138 mEq/L (IQR: 99–186 mEq/L), and the median salt consumption per person per day was 9.6 g (IQR: 7.3–14.5 g). According to the 24 h urine sodium content, the median salt consumption per person per day was 8.3 g (IQR: 6.0–10.3 g)

The median thyroid volume was 8.9 mL (IQR: 6.6–12.4 mL). Mean TSH, T4, and T3 were 3.72 ± 9.55 mIU/L, 8.99 ± 1.92 μg/dL, and 138.2 ± 20.12 ng/dL, respectively. Out of all subjects, 19 (18.4%) had thyroid nodules and 12 (11.7%) had subclinical hypothyroidism. These individuals were followed up by the endocrinologist.

The median UIC concentrations and the statistical differences between each characteristic are presented in Table 1.

**TABLE 1:** Urine iodine concentrations in the study participants based on characteristics.

		*N*	UIC Median (μg/L) (IQR)	*P* value
Sex	Male	24	188 (150 – 268.8)	0.405
	Female	68	175 (108 – 247)	
Hypertension	No	72	188 (133 – 258.8)	0.016∗
	Yes	20	133 (71 – 184.8)	
Salt storage	Out of sunlight	45	175 (102.5 – 244)	0.845
	In sunlight	12	166.5 (83 – 250)	
Thyroid nodule or goiter	No	57	188 (102.5 – 257.5)	0.286
	Yes	24	175 (133 – 208.3)	
Education level	Below high school	46	150 (79 – 225)	0.009∗
	High school graduate	39	213 (150 – 275)	
	College graduate or higher	7	184 (150 – 275)	
Salt added at the cooking stage	Before food preparation	56	186 (119 – 262.5)	0.286
	During food preparation	20	150 (83 – 225)	
	After food preparation	16	179 (133 – 231)	
Subclinical hypothyroidism	No	81	175 (111 – 250)	0.909
	Yes	11	183 (117 – 213)	
Thyroid volume, mL	<7	24	225 (150-271)	0.147
	7-10	24	175 (104-253)	
	>10	32	162.5 (83-213)	

∗Indicates statistically significant difference

No significant differences in UIC were observed regarding gender, storage method, presence of goiter or thyroid nodules, salt added by the cooking stage, or subclinical hypothyroidism. Subjects with hypertension or lower education levels had significantly lower iodine concentrations.

UIC was significantly positively correlated with urine sodium and TSH concentrations (correlation coefficient: 0.599, 0.208; *P* value: <0.001, 0.046, respectively) and significantly negatively correlated with T4 and thyroid volume (correlation coefficient: -0.245, -0.244; *P* value: 0.018, 0.029, respectively). There was a nonsignificant correlation between UIC and salt consumption per day.

**FIGURE 1:**
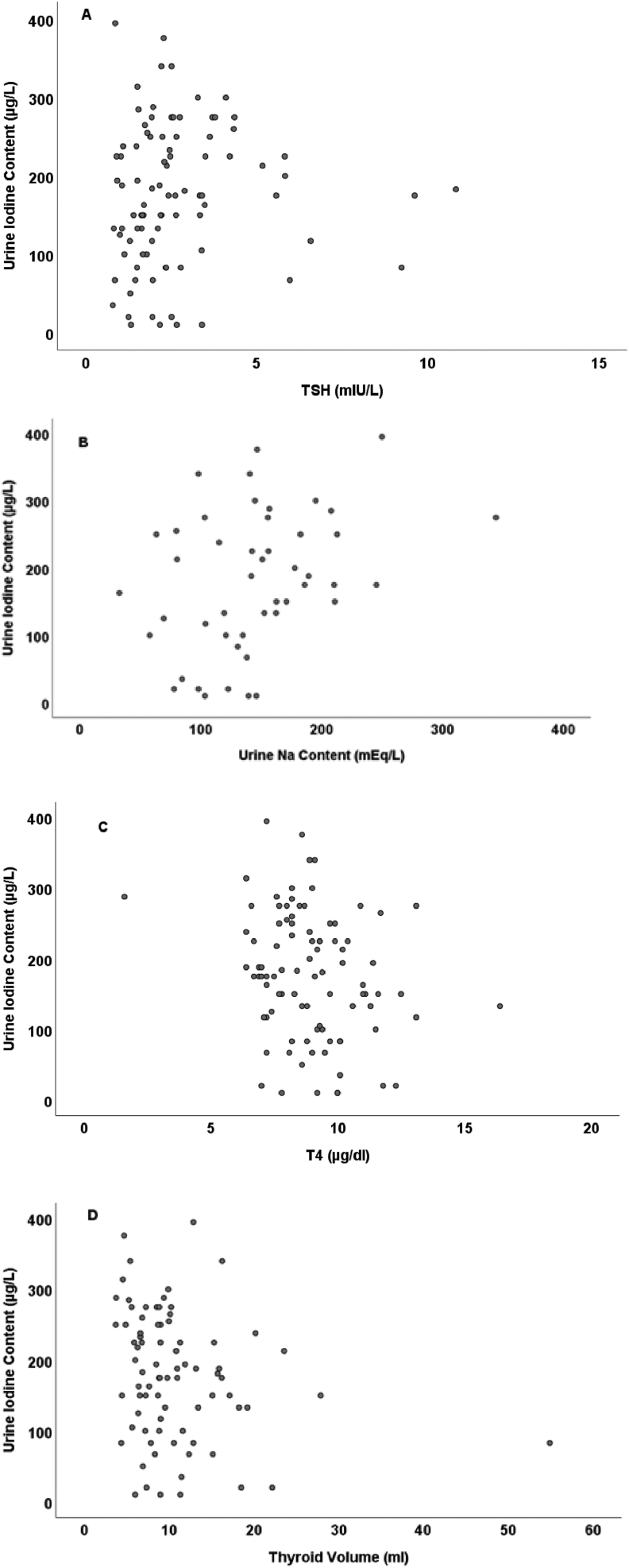
Scatter plot of urine iodine content with TSH (A), urine sodium content (B), T4 (C), and thyroid volume (D) in subjects.

Figure 1 illustrates the scatter dot graphs between UIC and urine sodium, thyroid volume, TSH, and T4 concentrations.

Median daily salt consumption in subjects with UIC of ≥100 μg/L was 10.31 g (IQR: 7.33–14.96 g; *N* = 66), whereas in those with UIC of <100 μg/L, it was 8.68 g (IQR: 7.12–12.04 g; *N* = 18). The difference was not statistically significant (*P* = 0.212).

Quantile regression indicated that age and urinary sodium concentration significantly predicted the level of UIC. Table 2 demonstrates the predictors for change in median urinary iodine concentration among the study subjects.

**TABLE 2:** Predictors of median urinary iodine concentration (μg/L).

Predictor variables	Number	Adjusted coefficient (95% CI)^1^	R^2^	*P* value
Age, y	86	-2.23 (-3.77, -0.69)	0.06	0.005∗∗
Urinary sodium concentration, mEq/L	43	0.86 (0.22, 1.50)	0.22	0.009∗∗
Thyroid volume, mL	74	-2.09 (-5.70, 1.50)	0.09	0.24
TSH, mIU/L	86	2.05 (-0.24, 4.39)	0.09	0.079
T4, μg/dL	86	-10.08 (-21.99, 1.82)	0.10	0.09
Salt used per day, g	78	2.46 (-2.41, 7.33)	0.07	0.31

1 The adjusted model including gender for age prediction, age, and gender for the other predictors. CI, confidence interval. ∗∗Indicates statistically significant difference.

## Discussion

The median UIC in the adult population in the city of Sadra was 175 μg/L, which categorizes the iodine status of the population as adequate based on WHO guidelines [5]. The results of our study were significantly higher than the 70 μg/L reported in adults (72.9 μg/L and 68.9 μg/L in men and women, respectively) and the 87.3 μg/L in the pregnant women population of Tehran [9, 10]. The median UIC in this study was similar to the 200 μg/L and 186.1 μg/L reported in 8–10-y-old school children of Shiraz (capital of Fars province) and Iran, respectively [11]. Median UIC values in adults worldwide range from 144 μg/L in China [15], 110 μg/L in the US [16], 46 μg/L in Italy [17], and 124.66 μg/L in Spain [18]. Results from our study indicated that national efforts to curb iodine deficiency were still successful 28 y after the introduction of the 1994 Salt Iodization Act.

Despite worldwide efforts to combat iodine deficiency, many countries have experienced decreased median UIC concentrations, while some have experienced a resurgence of iodine insufficiency. Median UIC has decreased from 133 μg/L in 2010 to 110 μg/L among US women of reproductive age, and from 320 μg/L in 1974 to 133 μg/L in the general US population in 2013 [16]. Previously iodine-sufficient countries such as Norway, Finland, and Germany have been categorized as iodine insufficient based on the latest IGN report [6]. Iodine insufficiency has also reoccurred in the past in France, Australia, and the United Kingdom, due to dietary changes [1, 19, 20]. In Columbia and Argentina, a decrease in the UIC concentrations has been attributed to a lack of proper iodine status monitoring and public health strategies [10, [21], [22], [23], [24]).

Studies in Iran have also shown a reduction in UIC concentrations [10]. Median UIC in adults of Tehran has dropped from 232 μg/L in 1996 to 100 μg/L in 2010 [10]. Similar results have also been reported in Tehrani pregnant women (186 μg/L in 1998 to 87.3 μg/L in 2014) and Iranian school children (205 μg/L in 1996 to 161 μg/L in 2013) [8, 9, [25], [26], [27]). Nazeri et al. [10] believe that this could be attributed to the reduction in salt consumption in Iran. The per capita salt intake in Tehran dropped from 10 g/d in 2000 to 8.4 g/d in 2009 [10], with similar results seen in Yazd and Isfahan [26]. Babaali et al. [27] found that the daily consumption of salt in Shiraz was 7.1 g. In this study, we found a much higher median salt intake of 9.59 g/d in adults of Sadra city. This number is much closer to the results observed in 2000 on Tehrani adults [10]. The higher salt consumption in Sadra can be a contributing factor to the higher UIC concentrations among the population. Moreover, most of the salt consumed by this study population was prepared in local factories from the salt mountains of Fars province. The different compositions of this salt may be involved in higher UIC among the population under study.

In this study, we found no significant difference in iodine concentrations between the sexes. Similar results were found by Nazeri et al. [10]. However, in studies by Iacone et al. [17] and Tayie et al. [28], men had higher UIC concentrations. We found a significantly negative correlation between hypertension and UIC concentrations. Similar results were found by Menon et al. [29] Meanwhile, Tayie et al. [28] found no correlation between UIC concentrations and high blood pressure, while Liu J et al. found a positive correlation between these factors [30]. Also, we observed that better education levels were associated with significantly higher UIC concentrations. Similar results were reported in Tehran by Nazeri et al. [10] using univariate logistic regression. However, this statistical significance was diminished when adjusting for household salt-iodine content and daily salt intake, suggesting that these factors were interconnected when leading to lower UIC concentrations [10]. Based on these results, we hypothesize that subjects with higher education levels are more likely to use iodized salt.

In this study, we found a positive correlation between TSH and UIC concentrations and a negative correlation between T4 and UIC concentrations, which contrasts with the negative correlation seen between these 2 factors, especially in iodine-deficient areas [31]. Previous studies showed no association between UIC and serum TSH or T4 [32]. Data from the national health and nutrition examination survey (NHANES) III indicated that higher urinary iodine excretion was significantly related to higher TSH concentration [33]. Although individuals with higher concentrations of urinary iodine excretion in our study had higher TSH and lower T4 concentrations, these concentrations were within the physiological range. The right-shift in the distribution of TSH in iodine-sufficient areas could be affected by hereditary and genetic influences on the set point of thyroid hormones [34].

During food preparation, some methods such as heating and washing can decrease the amount of salt-iodine content. Microwaving, boiling, washing, and poor salt preservation conditions including storage in sunlight can decrease the effective iodine content [35]. The WHO estimates a salt-iodine loss of ∼20% from retail until the food is served on the table [5]. Nevertheless, we found no significant difference in UIC between subjects who stored salt in the dark and those who did so in direct sunlight. Moreover, there was no difference in UIC between subjects that added salt before food preparation, during food preparation, or after the food was fully prepared.

We found no significant correlation between daily per capita salt intake and UIC concentrations. However, we found a significant positive correlation between UIC and urine sodium content, which is a more accurate method for measuring salt consumption [14, 36]. Similar results were found by Nazeri et al. [10]. These results along with those of other studies [10, 37, 38] and the WHO declaration [5] illustrate the link between salt consumption and iodine status. This can also further link the higher UIC concentrations found in Sadra city and the higher salt consumption than the results from Tehran [10].

In this study, we found a negative correlation between thyroid volume and UIC. Iodine deficiency has a goitrogenic effect and this negative correlation was anticipated. Some previous studies [39, 40] explained that the association between iodine status and thyroid volume was inconsistent; there was no correlation between thyroid volume and UIC in iodine-sufficient areas. The volume of the thyroid gland may be population-specific and some genetic and environmental factors may contribute to variations in thyroid volume, especially in iodine-sufficient areas [41].

This study has some limitations. The first limitation is its cross-sectional design and limited population in one city in the Fars province of Iran. Another possible limitation is that the iodine concentrations in the salt used by these study subjects were not measured.

In conclusion, iodine status in the adult population in the city of Sadra was categorized as sufficient, whereas it was insufficient in the adult and pregnant women populations of Tehran, the capital city of Iran. A significant contributing factor can be the higher use of salt among the population of Sadra than that of Tehran. High iodine concentrations in the environment, such as water and earth, can also hypothetically influence the iodine status. Studies with larger sample sizes and in other parts of the country are warranted.

## Author disclosures

MJ, AH, AS, no conflicts of interest.

## Data availability

Data described in the manuscript, codebook, and analytic code will be made available upon request to AS pending application and approval.

## Acknowledgments

We thank the Director and staff of the Department of Health at the Shiraz University of Medical Sciences for their time, effort, and contribution to this study; the staff of the Center for Development of Clinical Research of Nemazee Hospital for technical assistance; and Dr. Nasrin Shokrpour for editorial assistance. All authors: contributed to the study conception and design; MJ and AH: material preparation, data collection, and analysis; AH and MJ: drafted the first draft of the manuscript; and all authors: read and approved the final manuscript.

## Funding

The authors reported no funding received for this study.

## Appendix A Supplementary data

Multimedia components 1 Multimedia components 1
