# Supplementary material for: Sustained Elimination of Iodine Deficiency Within the Third Decade After Compulsory Iodine Supplementation Policy in the South of Iran: A Population-Based Cross-Sectional Study
Source: Curr Dev Nutr. 2022 Dec 22;7(1):100013. doi: 10.1016/j.cdnut.2022.100013 (PMC10100933; doi:10.1016/j.cdnut.2022.100013)
Supplement: Multimedia components 1 [file mmc1.pdf]

Questionnaire of the research project "Evaluation of iodine sufficiency in people over 18 years old in Sadra city in 2020

Code:		
Name:	Family Name:	Father’s Name:
Gender	Female	Male
Phone Number:		
Mobile Number:		
Address:		
<br>		
Education:<br><br>Below high school<br><br>High school graduate<br><br>College graduate<br><br>Advanced degree graduate		
The number of family members:<br><br>The number of family members at the time of consumption of iodized salt delivered:		

<br>		
Do you have past history of any medical problem?		
Hypertension	Yes	No
Diabetes Mellitus	Yes	No
Cardiovascular Disease	Yes<br>Disease Specification	No
Renal Disorder	Yes<br>Disease Specification	No
Gastrointestinal Disorder	Yes<br>Disease Specification	No
Thyroid Disorder	Yes<br>Disease Specification	No
Do you take any special medication?		
Anti-hypertensive	Yes<br>What drug?	No
Glucose lowering	Yes<br>What drug?	No
Lipid lowering	Yes	No

<table>
<tr><td></td><td>What drug?</td><td></td></tr>
<tr><td>Methimazole</td><td>Yes</td><td>No</td></tr>
<tr><td>Levothyroxine</td><td>Yes</td><td>No</td></tr>
<tr><td>Iron supplement</td><td>Yes</td><td>No</td></tr>
<tr><td>Iodine supplement</td><td>Yes</td><td>No</td></tr>
<tr><td>Calcium supplement</td><td>Yes</td><td>No</td></tr>
<tr><td>Zinc supplement</td><td>Yes</td><td>No</td></tr>
<tr><td colspan="3">**</td></tr>
<tr><td colspan="3">Do you smoke?
Yes …………Pack-year
No</td></tr>
<tr><td colspan="3">**</td></tr>
<tr><td>How many types of salt do you use at home?</td><td>One type</td><td>Several types
Type 1:

Type 2:

Type 3:</td></tr>
</table>

How do you store salt at home?<br><br>In a sealed container:<br><br>In a transparent bag:<br><br>In a cloudy bag:<br><br>Inside the closet away from sunlight:<br><br>Exposed to sunlight:
****
When do you add salt to food?<br><br>At the beginning of cooking<br><br>During cooking<br><br>At the end of cooking<br>**

Weight:	
Height:	
Waist circumference:	
Blood Pressure1:	
Blood Pressure2:	
The amount of salt delivered (grams):	The amount of salt withdrawn after two weeks (grams):

Amount of salt consumed per day (grams):	one person:	Whole family:

Name and surname of the questioner:

Questionnaire completion date:
